# Hippocampal mTOR Dysregulation and Morphological Changes in Male Rats after Fetal Growth Restriction

**DOI:** 10.3390/nu14030451

**Published:** 2022-01-20

**Authors:** Charlotte Schömig, Laura Oberholz, Gregor Fink, Jenny Voggel, Maria Wohlfarth, Jörg Dötsch, Kai-Dietrich Nüsken, Eva Nüsken

**Affiliations:** Department of Pediatrics, Faculty of Medicine and University Hospital Cologne, University of Cologne, 50937 Cologne, Germany; charlotte.schoemig@uk-koeln.de (C.S.); laura.oberholz@uk-koeln.de (L.O.); gregor.fink@uk-koeln.de (G.F.); jenny.voggel@uk-koeln.de (J.V.); maria.wohlfarth@uk-koeln.de (M.W.); joerg.doetsch@uk-koeln.de (J.D.); eva.nuesken@uk-koeln.de (E.N.)

**Keywords:** FGR, IUGR, low-protein diet, placental insufficiency, intrauterine stress, mTOR signaling, neurocognitive development, perinatal programming

## Abstract

Fetal growth restriction (FGR) has been linked to long-term neurocognitive impairment, especially in males. To determine possible underlying mechanisms, we examined hippocampal cellular composition and mTOR signaling of male rat FGR offspring during main brain growth and development (postnatal days (PND) 1 and 12). FGR was either induced by a low-protein diet throughout pregnancy, experimental placental insufficiency by bilateral uterine vessel ligation or intrauterine stress by “sham” operation. Offspring after unimpaired gestation served as common controls. Low-protein diet led to a reduced cell density in the molecular dentate gyrus subregion, while intrauterine surgical stress was associated with increased cell density in the cellular CA2 subregion. Experimental placental insufficiency caused increased mTOR activation on PND 1, whereas intrauterine stress led to mTOR activation on PND 1 and 12. To determine long-term effects, we additionally examined mTOR signaling and Tau phosphorylation, which is altered in neurodegenerative diseases, on PND 180, but did not find any changes among the experimental groups. Our findings suggest that hippocampal cellular proliferation and mTOR signaling are dysregulated in different ways depending on the cause of FGR. While a low-protein diet induced a decreased cell density, prenatal surgical stress caused hyperproliferation, possibly via increased mTOR signaling.

## 1. Introduction

Fetal growth restriction (FGR) is defined as “the failure of the fetus to reach its growth potential” [1]. While in developing countries FGR is typically caused by malnutrition, the most common cause in North America and Europe is placental insufficiency [2,3,4]. Aside from premature birth, FGR is the main cause of low birth weight, which is an important marker for perinatal morbidity and mortality [3,5]. Further studies showed that the adverse perinatal factors causing FGR also lead to long-term negative consequences. Apart from the augmented risk for metabolic, cardiovascular and renal diseases [6,7], FGR is associated with long-term neurocognitive impairment [8,9,10]. Children with FGR have a higher risk for deficiencies in learning, memory and attention as adolescents and adults compared to matching control groups [11,12,13,14,15,16,17]. Previous studies also linked low birthweight with a higher probability of age-related cognitive impairment and dementia in adulthood [18]. Males seem to be especially at risk to develop FGR-associated neurocognitive impairment in later life [19].

Cognitive impairment has also been shown in experimental FGR models in rats [20]. To study the FGR-associated consequences of maternal malnutrition, a model of maternal protein restriction during gestation is widely recognized [4,21]. A model of bilateral uterine artery and vein ligation has been well established to induce uteroplacental insufficiency in rats [4,21]. Previous studies showed that “sham” operation induces increased maternal corticosterone levels [22] as well as moderate FGR and programming in the offspring and therefore might be used as model for intrauterine stress [23].

Typical long-term neurocognitive consequences associated with FGR are deficiencies in learning and memory [17,20]. The hippocampus plays a central role in memory consolidation [24]. It is also known to be especially vulnerable to developmental and environmental influences [25,26,27]. Previous studies linked neurological long-term impairments of FGR to reduced hippocampal volume [28] and changes in cellular composition [29], expression of genes as well as epigenetic determinants [30,31,32]. However, the exact mechanisms of intrauterine programming of neurological consequences of FGR remain largely unknown [33].

Mammalian/Mechanistic Target of Rapamycin (mTOR) is a Serine/Threonine kinase, which is involved in cellular growth and metabolism through activation of anabolic and inhibition of catabolic processes. One of the best-studied processes regulated by mTOR is protein synthesis through phosphorylation of eukaryotic translation initiation factor 4E (eIF4E)-binding protein 1 (4E-BP1) and S6 kinase 1 (S6K1) [34,35]. Both are translational regulators. The function of 4E-BP1, which is to bind the cap-binding protein eIF4E, is inhibited by phosphorylation. As a result, the eIF4E complex can be formed and cap-dependent translation is possible. Phosphorylation of S6K1 increases protein synthesis, mRNA splicing, transcription, cell survival as well as cytoskeleton organization through its interaction with different targets. There are different isoforms of S6K1; the most studied form is p70 S6 kinase (p70S6K) [36].

In the central nervous system, mTOR plays an important role for brain development [37,38,39,40]. Reduction in mTOR activity may lead to neuronal degeneration, whereas mTOR hyperactivity may cause abnormal neuron and glia cell development and, subsequently, brain malformation [41]. It is assumed that mTOR activation through mTOR phosphorylation in the hippocampus plays an important role in spatial learning and short-term memory [42]. Previous studies also indicate a connection between mTOR hyperactivity and formation of abnormally hyperphosphorylated Tau protein, which is associated with neurodegenerative diseases such as Alzheimer’s disease [43,44,45]. Therefore, dysregulation of mTOR could be a possible explanation for long-term neurocognitive impairment in association with FGR.

This study was designed to test the hypothesis of whether dysregulation of mTOR signaling and altered cellular composition are common hippocampal signatures during early brain development after FGR of different origins. For this purpose, hippocampal tissue from FGR offspring after (1) low-protein (LP) diet throughout pregnancy, (2) bilateral uterine vessel ligation (LIG) during terminal pregnancy and (3) intrauterine stress (IUS) due to surgery without ligation (i.e., “sham” operation) in late pregnancy, each compared to an unimpaired control (C) group, was studied during brain development in early postnatal life (postnatal days (PND) 1 and 12) and adulthood (PND 180). Since previous studies showed that neurological deficits and hippocampal changes after FGR are particularly expressed in males [46,47,48], this study focused on male offspring.

## 2. Materials and Methods

### 2.1. Animals and Surgical Procedures

All animal procedures were conducted in accordance with the German regulations and legal requirements. The experimental protocol was approved by institutional and governmental review boards (LANUV NRW AZ 84-02.04.2012.A316).

In this study, male offspring from three rat models causing experimental FGR were compared to a common control (C) offspring group after unimpaired pregnancy of their dam (i.e., no surgery or special diet during gestation). FGR was induced either by low-protein (LP) diet of the dam, or bilateral uterine artery ligation (LIG) on gestational day (GD) 18, or intrauterine stress (IUS) by “sham” operation on GD 18 as described before [49]. As the focus of this study was to evaluate the effect of FGR on brain development and the hippocampus, and not to evaluate the effect of reduced uteroplacental blood flow itself, we refer to the offspring after “sham” operation of the dam as intrauterine stress (IUS) group. We did not compare LIG to IUS offspring.

Time-mated female Wistar rats were purchased from the research animal provider Janvier. Dams of the groups C, LIG and IUS received a normal protein diet throughout the study, dams of group LP received LP diet throughout pregnancy (i.e., from gestational day E0 until birth) and normal protein diet thereafter as described before [49]. Normal protein (NP) diet (Altromin C1000) is a standard rat maintenance diet containing 17% protein, while LP diet (Altromin C1003) contains 8.8% protein. Contents of energy (NP 3500 kcal/kg; LP 3262 kcal/kg), fat (NP 5%; LP 6.1%), disaccharides (11%), starch (NP 47%; LP 48%), methionine (NP 10 mg/kg; LP 8.7 mg/kg), folate (10 mg/kg) and the ratio of other amino acids, sodium (0.02%), vitamins and minerals were similar. In case of surgery, procedures were performed on gestational day (GD) 18. LIG and IUS dams were anesthetized with inhalative isoflurane 2.5% and received subcutaneous metamizole (100 mg/kg) for analgesia. After midline laparotomy both uterine horns were carefully placed out of the abdomen and a ligation of both uterine arteries and veins at the most caudal point accessible was performed in the LIG dams using 6-0 Prolene (Ethicon). IUS dams underwent identical anesthesia and surgery procedures, but without uterine vessel ligation. Duration of the whole procedure was 15–20 min per dam.

All pups were born spontaneously on GD 21 or GD 22 within a period of 12 h. To guarantee standard conditions, only litters between nine and 15 pups (mean litter size was expected to be 12) were included in the study. Pups were cross-fostered in pairs of two males and two females of every original litter whenever possible (C pups to other C foster-dams, LP pups to other LP foster-dams, LIG and IUS pups to C foster-dams) to minimize potential influences on brain development through maternal care and raised in groups of eight animals (four females and four males from two different litters). C, LP and IUS pups were chosen randomly for fostering. LIG pups were chosen randomly from the 6 smallest pups of the original litter for fostering. The rationale for preselection of LIG pups was comprehensively described before [49]. Only male pups were included into further analysis in this study. Half of the offspring was designated for molecular analyses, and the other half for histological analyses. On postnatal days (PND) 1 (C, *n* = 20 from 10 litters; LP, *n* = 16 from 8 litters; LIG, *n* = 16 from 10 litters; IUS, *n* = 20 from 10 litters), PND 12 (C, *n* = 16 from 8 litters ; LP, *n* = 15 from 8 litters; LIG, *n* = 13 from 7 litters ; IUS, *n* = 16 from 9 litters) and PND 180 (C, *n* = 12 from 7 litters; LP, *n* = 4 from 2 litters; LIG, *n* = 6 from 4 litters; IUS, *n* = 7 from 4 litters) the offspring were anesthetized using pentobarbital (5 mg/kg), perfused with NaCl 0.9% and decapitated. Afterwards, the brain was removed within 30–60 s. For molecular analyses, the brain was immediately transferred to ice cold artificial liquor and cut in the midline. Both halves of the hippocampus were obtained within 30 s, shock frozen in liquid nitrogen and stored at −80 °C. For histological analyses, brains of other offspring (from the same litters whenever possible) were immediately transferred to 4% paraformaldehyde (PFA). As especially brains on PND 1, but also on PND 12, were very vulnerable depending on time until transfer to PFA, we decided to restrict weighing to a small number of brains originally intended for histology (PND 1: C, *n* = 4 from 4 litters; LP, *n* = 7 from 7 litters; LIG, *n* = 4 from 3 litters; IUS, *n* = 3 from 3 litters; PND 12: C, *n* = 4 from 4 litters; LP, *n* = 7 from 7 litters; LIG, *n* = 6 from 6 litters; IUS, *n* = 7 from 7 litters). All molecular and some histological analyses were performed in tissue from brains not weighed before.

### 2.2. Protein and RNA Isolation

For protein isolation, the whole hippocampal tissue of one hemisphere either obtained on PND 1 or PND 12 was lysed in protein extraction buffer (10 mM Tris, pH 6.8; 6.65 M urea; 10% glycerol; 1% sodium dodecyl sulfate; 5 mM dithiothreitol; 0.5 mM phenylmethylsulfonyl fluoride), the tissue was homogenized by sonication (3 × 10% cycle, 50% energy), incubated on ice (1 h), and centrifuged (1600× *g*, 5 min, 4 °C). For isolation of protein from hippocampal tissue obtained on PND 180, the NucleoSpin^®^ RNA/Protein Kit (Machery-Nagel GmbH & Co. KG, Düren, Germany) was used. Protein concentrations were determined using the BCA protein assay kit (Thermo Scientific^TM^, Waltham, Massachusetts, USA).

### 2.3. Western Blot Techniques

We randomly selected five male rats representing five different litters per group and day for molecular studies on PND 1, PND 12 and PND 180 whenever possible. However, only four rats from two litters remained in the LP group on PND 180, and the five rats included from groups LIG and IUS on PND 180 represented four litters only. For protein detection, 30 µg protein from each hippocampal probe were separated on 12% acrylamide SDS-PAGE gels and transferred onto a nitrocellulose membrane for 120 min at 1.25 mA/cm^2^ using a Towbin buffer. Membranes were blocked with 5% milk and 2% BSA in Tris-buffered saline containing 0.05% Tween 20 (TBST) and subsequently incubated in the primary antibody (Table 1) at 4 °C overnight. After being rinsed with TBST and incubated with the secondary antibody (anti-rabbit IgG HRP-linked antibody, Cell Signaling #7074) for one hour at room temperature, they were developed using Amersham ECL Plus Solution (GE Healthcare, Little Chalfont, UK) and the ChemiDoc™ MP Imaging System (Bio-Rad Laboratories Inc., Hercules, California, USA). Western blots were performed to determine the amount of phosphorylated and total protein amounts of mTOR and its downstream proteins p70 S6 kinase (p70S6K) as well as eukaryotic initiation factor eIF4E-binding protein 1 (4E-BP1). Protein amounts were quantified through densitometry using Image Lab^TM^ (Bio-Rad Laboratories Inc., Hercules, California, USA). Proteins of interest were normalized to GAPDH. The phosphorylated protein to total protein ratio was determined to measure protein activation (mTOR, p70S6K) or inactivation (4E-BP1). On PND 180, we additionally examined expression and phosphorylation of Tau protein.

### 2.4. Histology

Brains from randomly selected males from each group (single males representing separate litters) on PND 1 (C, *n* = 5; LP, *n* = 5; LIG, *n* = 4; SOP, *n* = 5) and PND 12 (C, *n* = 5; LP, *n* = 7; LIG, *n* = 6; SOP, *n* = 7) were used for histological studies. Brains from PND 180 animals were not included in histological analysis due to technical issues. To fixate the tissue, brains were stored in 4% paraformaldehyde (pH 7.4) for 24 h immediately after removal. Afterwards, all samples were transferred into 70% isopropanol at 4 °C. After dewatering and saturating with 100% isopropanol, they were embedded in paraffin.

Subsequently, 10 μm coronary sections were cut. The equivalent of −3.24 mm Bregma in adult rat brains were stained with hematoxylin eosin. The cuts were analyzed using transmitted light microscopy and the open access software ImageJ (https://imagej.nih.gov/ij/, accessed on 15 January 2021). The hippocampal area was calculated by freehand selection in the cut equivalent of −3.24 mm Bregma in adult rat brains. To study the effect of experimental IUGR on hippocampal cell densities, we compared the molecular CA1 subregion and the cell bands of the CA2 and CA3 subregions of the cornu ammonis as well as the molecular (MoGD) and granulated (GrDG) subregions of the dentate gyrus. Total cell densities in the different hippocampal regions were measured using ImageJ Fiji. Therefore, defined regions of interest (ROIs) were created in the cut equivalent of −3.24 mm Bregma in adult rat brains. In that defined area, grayscale values were measured to determine cell densities in different hippocampal regions. For each animal, measurements were performed at three replicates per hippocampal region. For this purpose, three identical ROIs were placed within the region of interest and data averaged from all three ROIs were used for further analysis. All data were normalized to the mean of group C.

### 2.5. Statistical Analysis

All datasets were tested for standard distribution. In a few datasets one outlier was identified by Grubb’s test and excluded. Analysis of weight data was performed by Mann–Whitney tests with Bonferroni-adjusted *p*-values. Global analysis of cell densities as well as protein concentrations (densitometric data from Western blots) was performed by nonparametric one-way ANOVA. Dunn’s multiple comparison test was performed afterwards for the comparisons LP—C, LIG—C, IUS—C. All data are shown as mean ± standard deviation (SD). All (adjusted) *p*-values < 0.05 were considered significant.

## 3. Results

### 3.1. Postnatal Body Weight Was Reduced in All FGR Offspring Groups, While Brain- to Body-Weight Ratios Were Elevated after Experimental Placental Insufficiency on PND 1 and after Low-Protein Diet as Well as after Intrauterine Stress on PND 12

**Figure 1:**
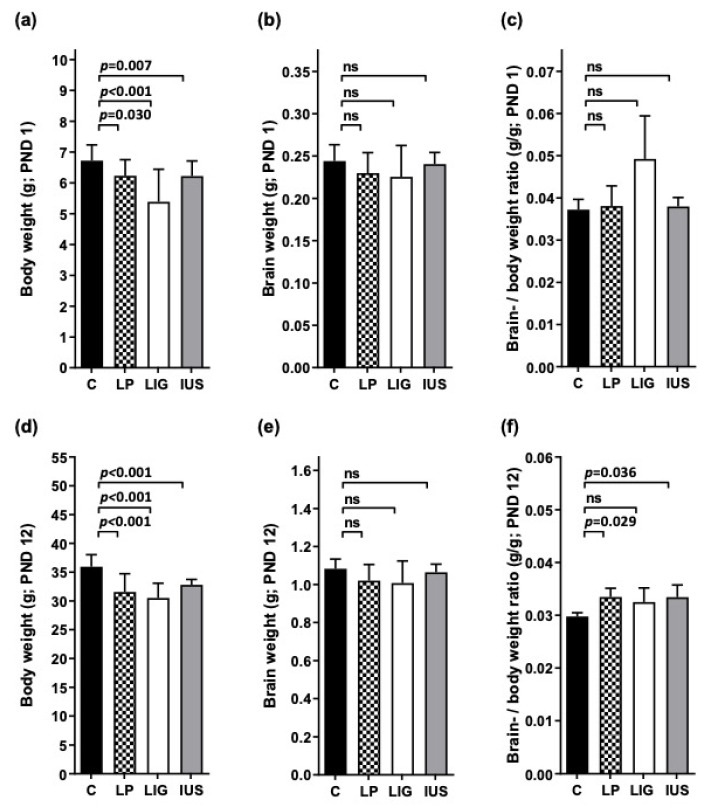
(**a**) Body weight, (**b**) brain weight and (**c**) brain- to body-weight ratio on postnatal day (PND) 1 as well as (**d**) body weight, (**e**) brain weight and (**f**) brain- to body-weight ratio on PND 12. For detailed offspring number information see methods section. C, control offspring after unimpaired gestation; LP, offspring after low-protein diet throughout gestation; LIG, offspring after bilateral uterine vessel ligation; IUS, offspring after intrauterine stress through “sham” operation. Values are expressed as mean ± standard deviation. In case of significance compared to C, adjusted *p*-values (Mann–Whitney tests) are indicated above the bars; ns, not significant.

On PND 1, weight data of the offspring included in this study resembled birth weight data of all offspring of the superordinate study published before [41] and were significantly reduced in all three FGR offspring groups (mean values of LIG < LP = IUS (Figure 1a)). Brain weight was not significantly altered in any of the FGR offspring groups (Figure 1b), and brain- to body-weight ratio was increased in LIG offspring (which was not significant due to the low number of available brain weights) but not in LP or IUS offspring (Figure 1c). On PND 12, body weight still was significantly reduced in all FGR offspring groups (Figure 1d), brain weight again was similar to controls (Figure 1e), and brain- to body-weight ratios were significantly increased in LP and IUS, but no longer in LIG offspring (Figure 1f). In the long term (PND 180), there were no significant differences in body weight among the different groups (C, 572 ± 35 g; LP, 600 ± 28 g; LIG, 538 ± 45 g; IUS, 605 ± 51 g). All groups showed similar brain weights (C, 2.21 ± 0.13 g; LP, 2.27 ± 0.05 g; LIG, 2.18 ± 0.06 g; IUS, 2.27 ± 0.05 g) and brain-weight to body-weight ratios (C, 0.39 ± 0.03 g/100 g; LP, 0.38 ± 0.02 g/100 g; LIG, 0.41 ± 0.02 g/100 g; IUS, 0.36 ± 0.04 g/100 g).

### 3.2. Hippocampal Area in Relation to Brain and Body Weight Was Augmented after Low-Protein Diet and Experimental Placental Insufficiency on PND 12

On PND 1, there were neither significant differences in hippocampal areas (C, 0.76 ± 0.19 mm^2^; LP, 0.65 ± 0.12 mm^2^; LIG, 0.60 ± 0.17 mm^2^; IUS, 0.70 ± 0.22 mm^2^) nor in hippocampal area to body weight (C, 0.11 ± 0.02 mm^2^/g; LP, 0.11 ± 0.02 mm^2^/g; LIG, 0.12 ± 0.02 mm^2^/g; IUS, 0.11 ± 0.04 mm^2^/g) between the offspring groups. On PND 12, LP and LIG offspring showed significantly larger hippocampal area in relation to body weight (C, 0.07 ± 0.01 mm^2^/g; LP, 0.13 ± 0.04 mm^2^/g; LIG, 0.11 ± 0.01 mm^2^/g; IUS, 0.09 ± 0.01 mm^2^/g; LP—C, *p* = 0.002; LIG—C, *p* = 0.007) compared to the control offspring group. Absolute values of the hippocampal area were not significantly different between the groups on PND 12 (C, 2.97 ± 0.54 mm^2^; LP, 3.00 ± 0.50 mm^2^; LIG, 3.16 ± 0.39 mm^2^; IUS, 2.88 ± 0.23 mm^2^).

### 3.3. Low-Protein Diet Led to Decreased Hippocampal Cell Density in the Molecular Dentate Gyrus Subregion While Intrauterine Stress Was Associated with Increased Cell Density in the Cellular CA2 Subregion

**Figure 2:**
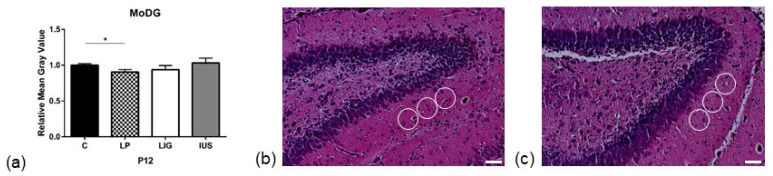
(**a**) Results of cell density analysis in the molecular dentate gyrus (MoDG) subregion of the hippocampus on postnatal day PND 12 (C, *n* = 5; LP, *n* = 7; LIG, *n* = 6; IUS, *n* = 6). For each animal, grayscale measurements were performed at three replicates per hippocampal region. All data were normalized to the mean of the control group (C). Representative images of cell density measurement of (**b**) control (C) offspring and (**c**) offspring after low-protein (LP) diet in the MoDG subregion of the hippocampus on postnatal day PND 12 (HE staining, 20-fold magnification). White circles indicate regions of interest (ROIs) used for quantitative analysis. Scale bar = 50 μm. Asterisks indicate significance; *, adjusted *p* < 0.05.

**Figure 3:**
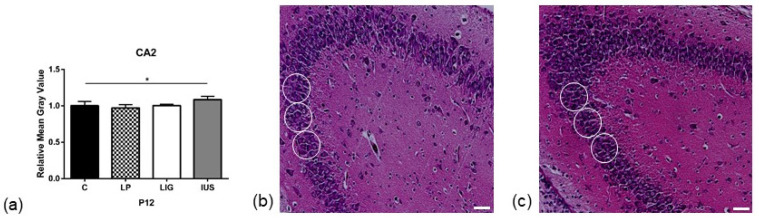
(**a**) Results of cell density analysis in the cellular CA2 subregion of the hippocampus on postnatal day PND 12 (C, *n* = 5; LP, *n* = 7; LIG, *n* = 6; IUS, *n* = 7). For each animal, grayscale measurements were performed at three replicates per hippocampal region. All data were normalized to the mean of the control group (C). Representative images of cell density measurement of (**b**) control (C) offspring and (**c**) offspring after intrauterine stress (IUS) in the cellular CA2 subregion of the hippocampus on postnatal day PND 12 (HE staining, 20-fold magnification). White circles indicate regions of interest (ROIs) used for quantitative analysis. Scale bar = 50 μm. Asterisks indicate significance; *, adjusted *p* < 0.05.

On PND 1, we did not find any significant alterations to cell densities in the hippocampal subregions studied (Table S1). On PND 12, we found a significantly decreased cell density in the molecular dentate gyrus (MoDG) hippocampal subregion of LP offspring (C, 1.00 ± 0.02; LP, 0.90 ± 0.04; LIG, 0.94 ± 0.06, IUS, 1.03 ± 0.07) (Figure 2, Table S1) as well as a significantly increased cell density in the cellular CA2 hippocampal subregion of IUS offspring (C, 1.00 ± 0.06; LP, 0.97 ± 0.04; LIG, 1.00 ± 0.02, IUS, 1.09 ± 0.05) (Figure 3, Table S1).

### 3.4. Experimental Placental Insufficiency Caused mTOR Activation on PND 1, Whereas Intrauterine Stress Caused mTOR Activation throughout Postnatal Hippocampal Development

**Figure 4:**
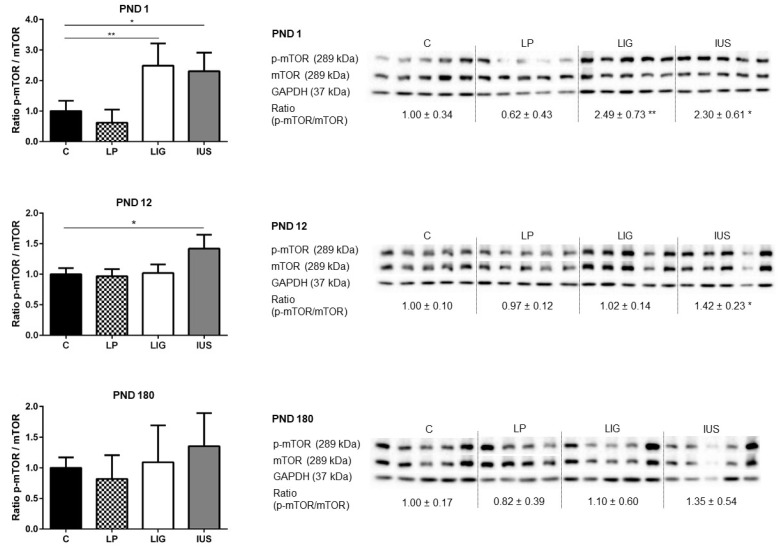
Western blot analyses of phospho-mTOR (p-mTOR, Ser2448) and mTOR proteins in the hippocampus of male rats on postnatal days PND 1, PND 12 (C, *n* = 5; LP, *n* = 5; LIG, *n* = 5; IUS, *n* = 5) and PND 180 (C, *n* = 5; LP, *n* = 4; LIG, *n* = 5; IUS, *n* = 5). Glyceraldehyde 3-phosphate dehydrogenase (GAPDH) was used as additional reference protein for quality assurance but not used for calculations. Densitometric ratios were calculated for p-mTOR/mTOR and are shown for each group (C, controls; LP, low protein; LIG, ligation; IUS, intrauterine stress) as mean ± SD directly below the appropriate Western blot signals. Data were compared by nonparametric one-way ANOVA. Dunn’s post-test was performed for the comparisons LP—C, LIG—C, IUS—C. Asterisks indicate significance; *, adjusted *p* < 0.05; **, adjusted *p* < 0.01. The ratio of p-mTOR/mTOR was significantly increased in LIG offspring on PND 1, as well as in IUS offspring on PND 1 and PND 12.

Western blot analysis revealed a significantly increased amount of phosphorylated mTOR (p-mTOR/GAPDH: C, 1.00 ± 0.49; LP, 0.96 ± 0.64; LIG, 1.79 ± 0.25; IUS, 2.12 ± 0.50) as well as significantly elevated p-mTOR/mTOR ratios in LIG and IUS but not LP pups compared to pups of group C on PND 1 (Figure 4). On PND 12, IUS pups still presented with an elevated p-mTOR/mTOR ratio in comparison to C (Figure 4). On PND 180, there were no significant alterations to mTOR phosphorylation in any of the FGR groups compared to the control group (Figure 4).

**Figure 5:**
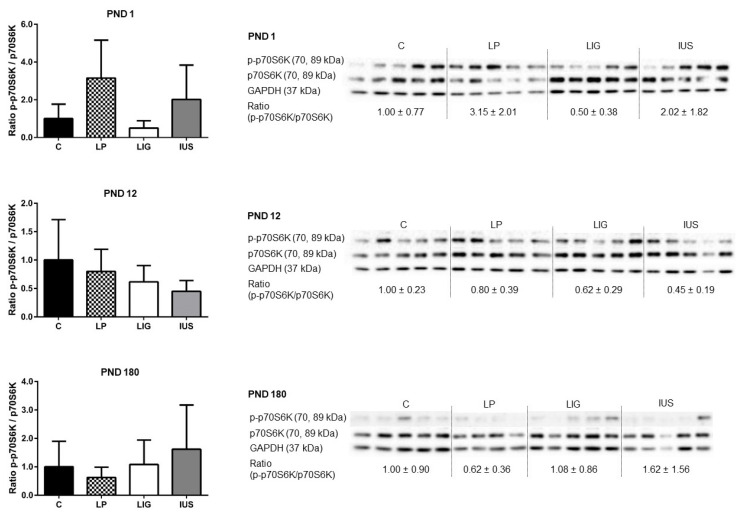
Western blot analyses of phospho-p70 S6 Kinase (p-p70S6K, Thr389) and p70S6K proteins in the hippocampus of male rats on postnatal days PND 1, PND 12 (C, *n* = 5; LP, *n* = 5; LIG, *n* = 5; IUS, *n* = 5) and PND 180 (C, *n* = 5; LP, *n* = 4; LIG, *n* = 5; IUS, *n* = 5). Glyceraldehyde 3-phosphate dehydrogenase (GAPDH) was used as additional reference protein for quality assurance but not used for calculations. Densitometric ratios were calculated for p-p70S6K/p70S6K and are shown for each group (C, controls; LP, low protein; LIG, ligation; IUS, intrauterine stress) as mean ± SD directly below the appropriate Western blot signals. Data were compared by nonparametric one-way ANOVA. Dunn’s post-test was performed for the comparisons LP—C, LIG—C, IUS—C. There were no significant differences between the groups.

**Figure 6:**
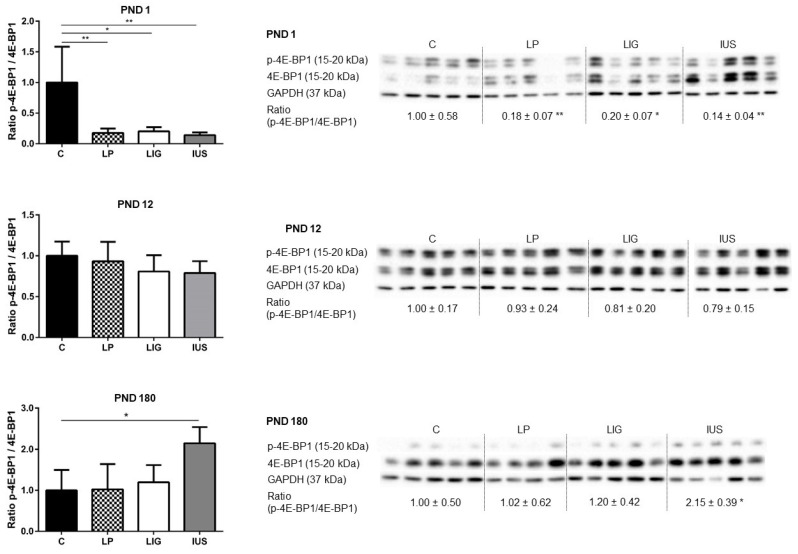
Western blot analyses of phospho-eukaryotic translation initiation factor 4E binding protein 1 (p-4E-BP1, Thr37/46) and 4E-BP1 proteins in the hippocampus of male rats on postnatal days PND 1, PND 12 (C, *n* = 5; LP, *n* = 5; LIG, *n* = 5; IUS, *n* = 5) and PND 180 (C, *n* = 5; LP, *n* = 4; LIG, *n* = 5; IUS, *n* = 5). Glyceraldehyde 3-phosphate dehydrogenase (GAPDH) was used as additional reference protein for quality assurance but not used for calculations. Densitometric ratios were calculated for p-4E-BP1/4E-BP1 and are shown for each group (C, controls; LP, low protein; LIG, ligation; IUS, intrauterine stress) as mean ± SD directly below the appropriate Western blot signals. Data were compared by nonparametric one-way ANOVA. Dunn’s post-test was performed for the comparisons LP—C, LIG—C, IUS—C. Asterisks indicate significance; *, adjusted *p* < 0.05; **, adjusted *p* < 0.01. The ratio of p-4E-BP1/4E-BP1 was significantly reduced in LIG, IUS and LP offspring on PND 1, as well as increased in IUS offspring on PND 180.

Looking at downstream mediators, LIG pups presented with a significantly higher expression of total p70S6K on PND 1 (p70S6K/GAPDH: C, 1.00 ± 0.40; LP, 0.86 ± 0.39; LIG, 1.68 ± 0.19, IUS, 1.13 ± 0.51) and PND 12 (p70S6K/GAPDH: C, 1.00 ± 0.17; LP, 1.36 ± 0.26; LIG, 1.58 ± 0.36; IUS, 1.29 ± 0.37). However, neither phosphorylated p70S6K (p-p70S6K/GAPDH), being the active mediator, nor p-p70S6K/p70S6K ratios were significantly different in any of the FGR groups compared to C (Figure 5). Total 4E-BP1, which is the active mediator inhibiting transcription, was significantly increased in IUS pups on PND 1 (4E-BP1/GAPDH: C, 1.00 ± 0.81; LP, 3.07 ± 1.93; LIG, 2.63 ± 1.30, IUS, 7.23 ± 3.85). The p-4E-BP1/4E-BP1 ratio was significantly decreased in LP, LIG as well as in IUS on PND 1 (Figure 6). On PND 12, there were no significant differences in 4E-BP1 protein expression or phosphorylation. On PND 180, the p-4E-BP1/4E-BP1 ratio was significantly increased in IUS in comparison to C (Figure 6).

### 3.5. Experimental Placental Insufficiency, Intrauterine Stress and Maternal Low-Protein Diet Did Not Have Long-Term Effects on Expression or Phosphorylation of Tau Proteins

**Figure 7:**
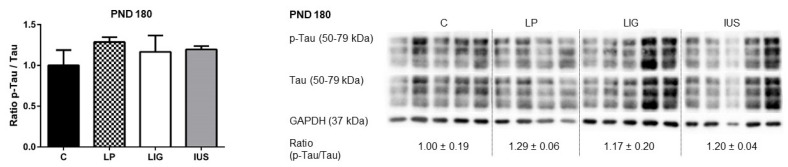
Western blot analyses of phospho-Tau (p-Tau, S396) and Tau proteins in the hippocampus of male rats on postnatal day PND 180 (C, *n* = 5; LP, *n* = 4 LIG, *n* = 5; IUS, *n* = 5). Glyceraldehyde 3-phosphate dehydrogenase (GAPDH) was used as additional reference protein for quality assurance but not used for calculations. Densitometric ratios were calculated for p-Tau/Tau and are shown for each group (C, controls; LP, low protein; LIG, ligation; IUS, intrauterine stress) as mean ± SD directly below the appropriate Western blot signals. Data were compared by nonparametric one-way ANOVA. Dunn’s post-test was performed for the comparisons LP—C, LIG—C, IUS—C. There were no significant differences between the groups.

Western blot analysis did not reveal any significant differences in the expression of hippocampal Tau protein in LP, IUS or LIG rats on PND 180. Furthermore, the level of Tau phosphorylation at S396 was similar among the different groups (Figure 7).

## 4. Discussion

Fetal growth restriction (FGR), which is most commonly caused by undernutrition and placental insufficiency, remains a highly relevant global health problem [2]. Neurocognitive short- and long-term consequences subsequent to impaired neuronal development including the hippocampus have been described after FGR [8,9]. The present study was designed to test the hypothesis that dysregulation of mTOR signaling during the period of maximal brain growth between birth and PND 12 and altered cellular composition are common hippocampal signatures after FGR of different origins in male rats. In our experimental setting, FGR was either induced by maternal low-protein (LP) diet, experimental utero-placental insufficiency due to bilateral ligation (LIG) of the uterine arteries and veins or by intrauterine stress (IUS) by “sham” operation during pregnancy. Our results demonstrate that cellular proliferation and mTOR signaling in the hippocampus are dysregulated in different ways depending on the cause of FGR.

As we have shown previously, our present data confirm that not only maternal low-protein diet and bilateral ligation of the Arteriae and Venae uterinae, but also intrauterine stress through prenatal surgical procedures in the dam caused significant global growth restriction in the pups [23]. All groups showed catch-up growth leading to similar body weights on PND 180. While all FGR groups showed constant postnatal growth restriction, absolute brain weight did not significantly differ from the control group. As a result, the brain- to body-weight ratio was significantly increased in all FGR offspring groups at some point of postnatal brain development. These data are in line with a phenomenon commonly known as brain sparing effect [2]. However, it remains controversial if brain sparing is a result of mechanisms protecting the brain or rather indicates pathology [50,51,52]. As we had hypothesized, we found significant alterations to cellular density in hippocampal tissue after FGR. Notably, these alterations were dependent upon the cause of FGR. While placental insufficiency did not have a significant impact and maternal low-protein diet caused decreased cell densities, perinatal stress seems to be associated with hyperproliferation. In addition, different hippocampal subregions are affected. This is in line with a previous study on maternal malnutrition which showed that different hippocampal subregions were affected differently depending on the composition of maternal nutrition [28].

In our LP group, we could demonstrate decreased cell densities in the molecular dentate gyrus (MoDG) subregion. The MoDG region is known to be crucial in the process of recalling contextual memories after several hours or longer [53]. Our findings indicate that a low-protein diet during brain development may particularly affect this region, with possible consequences for long-term memory. These results support previous studies. Thus, Berardino et al. and Ferroni et al. showed that a perinatal low-protein diet in mice was associated with memory deterioration in the offspring [54,55]. Wang et al. found impairment of spatial learning and memory associated with decreased hippocampal brain-derived neurotrophic factor (BDNF) levels in rats after maternal low-protein diet during pregnancy [56].

In contrast, IUS pups presented with increased hippocampal cell densities. Interestingly, we found significantly increased cell densities in the CA2 region, which was neither affected in LP nor LIG offspring. Thus, perinatal stress can be considered as an independent risk factor for region-specific changes in neuronal development [57,58,59,60]. Stress may also be a compensating factor with respect to hippocampal proliferation in situations impairing proliferation, such as placental insufficiency, which may explain the absence of significantly reduced cell densities in our LIG offspring group.

On the molecular level, the low-protein group did not show significant differences in mTOR activation in comparison to the control group. However, both experimental placental insufficiency as well as intrauterine stress induced increased hippocampal mTOR activation on PND 1. Since both LIG and IUS pups experience perinatal stress by surgery, surgical stress and post-operative recovery might explain common findings in these groups. Furthermore, the anesthetics used during maternal surgery may have had a neurotoxic effect. Several studies have shown that isoflurane may increase mTOR activity, which has been discussed as a possible underlying cause for anesthetic-induced neurotoxicity [61,62]. Interestingly, while mTOR activity of LIG offspring normalized on PND 12, IUS offspring presented with significant mTOR activation throughout early postnatal life. In the context of the earlier described changes in hippocampal cellular composition, these findings suggest that late increased mTOR phosphorylation may contribute to the observed increased cell density in the CA2 region. Pursuing this hypothesis could provide potential “re-programming” approaches using mTOR inhibitors such as rapamycin.

Since downstream results of mTOR signaling appear to be inconsistent, further studies are needed to clarify the exact mechanisms. We found increased concentrations of total p70S6K1 in LIG and of total 4E-BP1 in all experimental groups on PND 1. However, despite increased mTOR phosphorylation, there was no significant difference in phosphorylation of 4E-BP1 or p70S6K1 in LIG or IUS throughout early postnatal life. Previous studies have shown that 4E-BP1 and p70S6K1 are phosphorylated by other kinases besides mTOR [63,64,65], possibly explaining the differences in phosphorylation observed in this study.

Apart from neurodevelopmental aspects, previous studies have shown that hippocampal mTOR activation plays a role in spatial learning. Qi and colleagues compared hippocampal mTOR signaling in a learning group to a control group and found similar total amounts of the crucial proteins, but significantly increased amounts of phosphorylated proteins. Additionally, they described retardation of the learning process by infusion of rapamycin into the ventricle system [42]. Even if we could not provide evidence for differences in mTOR phosphorylation in adult FGR animals, this does not rule out functional dysregulation. Significantly increased 4E-BP1 phosphorylation in adult IUS offspring could also contribute to IUGR-associated hippocampal sequels through induction of protein translation. In our study, timing of sacrifice was not synchronized to learning tasks.

Additionally, when looking at late effects of mTOR activity, both human and experimental studies indicate a direct and indirect connection between mTOR hyperactivity and formation of abnormally hyperphosphorylated Tau protein, which is associated with Alzheimer’s disease [43,44,45]. Most interestingly, Caccamo and colleagues found an association between Alzheimer’s disease, such as cognitive impairment and mTOR hyperactivity, in a mouse model and additionally showed that genetic mTOR suppression leads to reduction of beta-amyloid plaques and cognitive deficiencies [66]. Streptozotocin induced diabetic mice also develop activation of mTOR and cognitive impairment [67]. The diabetes-associated cognitive deficiencies were eliminated by small doses of rapamycin by gavage [67]. In contrast, our study showed no long-term effects on mTOR phosphorylation, although FGR was associated with early postnatal changes in hippocampal mTOR activity. Correspondingly, we did not observe long-term changes of expression and phosphorylation of hippocampal Tau protein after FGR. Hence, we conclude that cognitive impairment in the context of FGR is mediated by mechanisms differing from the neurocognitive changes in tauopathies such as Alzheimer’s disease.

Our study has a couple of limitations. First, area measurements and histologic analyses were limited to global analysis of cell densities and were performed at a single Bregma level only. Immunofluorescence staining regarding mTOR signaling was not possible due to technical issues within the available thickness of brain cuts. Second, protein was isolated from total hippocampal tissue. Therefore, region-specific or cell-type specific protein dysregulation could not be analyzed. To support results regarding protein expression, mRNA expression analysis (e.g., Transcriptomics) could be included in future studies. A strength of our study is the longitudinal design comparing different causes of FGR.

We used different rat models to investigate different causes of fetal growth restriction. Previous studies have shown that, similar to humans, rats also develop neurocognitive impairment of learning and memory after FGR [20]. Males are affected more often [19,68]. Hippocampal changes associated with FGR have been found in both rat and human studies. Thus, reduced hippocampal volume has been demonstrated in MRI studies of children born small for gestational age [69,70] as well as in common experimental FGR rat models [28,30]. Although possible underlying molecular mechanisms in the hippocampus have been mainly studied in experimental animal models, there is reason to assume that the histological and molecular observations made in our study also might apply to humans to some extent. Nevertheless, as previous studies have pointed out, differences between species regarding the course of gestation, placental physiology, brain development as well as the severity and timing of the interventions in the experimental models need to be considered when interpreting the presented data and limit transferability to humans [31,71].

## 5. Conclusions

In conclusion, our findings provide evidence that cellular proliferation in the hippocampus and hippocampal mTOR signaling are dysregulated during postnatal brain development after FGR. Interestingly, the time course and the type of dysregulation depend on the cause of FGR. While a low-protein diet induces decreased cell densities, especially in the MoDG hippocampal subregion, prenatal stress by surgery in the dam causes hyperproliferation in the CA2 region, possibly via increased mTOR signaling. Basal hippocampal mTOR phosphorylation in adult FGR animals was unaltered while we did not test mTOR signaling during specific learning tasks.

## Figures and Tables

**Table 1:** Primary antibodies.

Antibody	Manufacturer	Molecular Weight	Dilution *
4E-BP1 Rabbit mAb	Cell Signaling Technology (#9644)	15–20 kDa	1:1000
Phospho-4E-BP1 (Thr37/46) Rabbit mAb	Cell Signaling Technology (#2855)	15–20 kDa	1:2000
GAPDH Rabbit mAb	Cell Signaling Technology (#2118)	20 kDa	1:3000
mTOR Rabbit mAb	Cell Signaling Technology (#2983)	289 kDa	1:1000
Phospho-mTOR (Ser2448) Rabbit mAb	Cell Signaling Technology (#5536)	289 kDa	1:500
p70S6K Rabbit mAb	Cell Signaling Technology (#2708)	70/89 kDa	1:500
Phospho-p70S6K (Thr389) Rabbit mAb	Cell Signaling Technology (#9234)	70/89 kDa	1:500
Tau Rabbit mAb	Abcam (ab32057)	50–79 kDa	1:2000
Phospho-Tau (Ser396) Rabbit mAb	Abcam (ab109390)	50–79 kDa	1:10,000

* in 5% BSA/TBST.

## Data Availability

The data presented in this research are available on request from the corresponding author.

## Supplementary Materials

The following supporting information can be downloaded at: https://www.mdpi.com/article/10.3390/nu14030451/s1: Table S1: Results of cell density measurements in hippocampal subregions on postnatal days 1 and 12.
