# Supplementary material for: Hippocampal mTOR Dysregulation and Morphological Changes in Male Rats after Fetal Growth Restriction
Source: Nutrients. 2022 Jan 20;14(3):451. doi: 10.3390/nu14030451 (PMC8839133; doi:10.3390/nu14030451)
Supplement: Supplementary file 1 [file nutrients-14-00451-s001.zip › nutrients-1547227-supplementary.pdf]

Table S1. Results of cell density measurements in hippocampal subregions on postnatal days 1 (C=5, LP=5, LIG=4, IUS=5) and 12 (C=5, LP=7, LIG=6, IUS=7). For each animal, grayscale value measurements were performed at three replicates per hippocampal region in the cut equivalent of -3,24 mm Bregma in adult rat brains. For this purpose, three identical ROIs were placed within the region of interest and data averaged from all three ROIs was used for further analysis. All data was normalized to the mean of group C.

Day of life	Region	Subregion	Cell density (fold change normalized to C)				KW-Test (*P*)	Dunn's post-test (*P*)		
			C	LP	LIG	IUS		LP – C	LIG – C	IUS – C
PND 1	CA1	molecular	1.00 ± 0.10	0.98 ± 0.05	1.04 ± 0.05	1.06 ± 0.04	0.260	0.999	0.999	0.532
	CA2	cellular	1.00 ± 0.03	0.99 ± 0.09	1.02 ± 0.05	1.07 ± 0.06	0.366	0.999	0.999	0.245
	CA3	cellular	1.00 ± 0.08	0.97 ± 0.06	1.06 ± 0.05	1.08 ± 0.07	0.070	0.999	0.684	0.310
	DG	molecular	n.d.	n.d.	n.d.	n.d.	n.d.	n.d.	n.d.	n.d.
	DG	granular	1.00 ± 0.05	1.01 ± 0.14	1.03 ± 0.10	1.09 ± 0.09	0.289	0.999	0.999	0.532
PND 12	CA1	molecular	1.00 ± 0.03	0.95 ± 0.04	0.98 ± 0.03	1.05 ± 0.05	0.005 (*)	0.247	0.999	0.445
	CA2	cellular	1.00 ± 0.06	0.97 ± 0.04	1.00 ± 0.02	1.09 ± 0.04	0.005 (*)	0.999	0.999	0.049 (*)
	CA3	cellular	1.00 ± 0.05	0.98 ± 0.04	1.01 ± 0.05	1.09 ± 0.05	0.004 (*)	0.999	0.999	0.061
	DG	molecular	1.00 ± 0.02	0.90 ± 0.04	0.94 ± 0.06	1.03 ± 0.07	0.004 (*)	0.024 (*)	0.306	0.999
	DG	granular	1.00 ± 0.05	0.97 ± 0.05	1.01 ± 0.01	1.05 ± 0.06	0.107	0.999	0.999	0.423

C, control offspring after unimpaired gestation; LP, offspring after low protein diet of the dam throughout gestation; LIG, offspring after bilateral uterine vessel ligation on gestational day (GD) 18; IUS, offspring after intrauterine stress by "sham" operation on GD 18; KW-Test, Kruskal-Wallis-test (nonparametric ANOVA); *P*, *P*-value; LP – C, comparison of LP and C offspring; LIG – C, comparison of LIG and C offspring; IUS – C, comparison of IUS and C offspring; PND, postnatal day; CA, cornu ammonis; DG, dentate gyrus; n.d., not detemined.
